# Analysis of the type II-A CRISPR-Cas system of *Streptococcus agalactiae* reveals distinctive features according to genetic lineages

**DOI:** 10.3389/fgene.2015.00214

**Published:** 2015-06-15

**Authors:** Clément Lier, Elodie Baticle, Philippe Horvath, Eve Haguenoer, Anne-Sophie Valentin, Philippe Glaser, Laurent Mereghetti, Philippe Lanotte

**Affiliations:** ^1^UMR1282 Infectiologie et Santé Publique, Bactéries et Risque Materno-Foetal, Université de Tours, ToursFrance; ^2^INRA, UMR1282 Infectiologie et Santé Publique, NouzillyFrance; ^3^Service de Bactériologie-Virologie, Hôpital Bretonneau – Centre Hospitalier Régional et Universitaire de Tours, ToursFrance; ^4^DuPont Nutrition and Health, Dangé-Saint-RomainFrance; ^5^Unité de Biologie des Bactéries Pathogènes à Gram Positif, Institut Pasteur, ParisFrance; ^6^CNRS UMR 3525, ParisFrance

**Keywords:** *Streptococcus agalactiae*, CRISPR-Cas, phylogeny, ST-17, typing

## Abstract

CRISPR-Cas systems (clustered regularly interspaced short palindromic repeats/CRISPR-associated proteins) are found in 90% of archaea and about 40% of bacteria. In this original system, CRISPR arrays comprise short, almost unique sequences called spacers that are interspersed with conserved palindromic repeats. These systems play a role in adaptive immunity and participate to fight non-self DNA such as integrative and conjugative elements, plasmids, and phages. In *Streptococcus agalactiae*, a bacterium implicated in colonization and infections in humans since the 1960s, two CRISPR-Cas systems have been described. A type II-A system, characterized by proteins Cas9, Cas1, Cas2, and Csn2, is ubiquitous, and a type I–C system, with the Cas8c signature protein, is present in about 20% of the isolates. Unlike type I–C, which appears to be non-functional, type II-A appears fully functional. Here we studied type II-A CRISPR-*cas* loci from 126 human isolates of *S. agalactiae* belonging to different clonal complexes that represent the diversity of the species and that have been implicated in colonization or infection. The CRISPR-*cas* locus was analyzed both at spacer and repeat levels. Major distinctive features were identified according to the phylogenetic lineages previously defined by multilocus sequence typing, especially for the sequence type (ST) 17, which is considered hypervirulent. Among other idiosyncrasies, ST-17 shows a significantly lower number of spacers in comparison with other lineages. This characteristic could reflect the peculiar virulence or colonization specificities of this lineage.

## Introduction

*Streptococcus agalactiae*, or group B *Streptococcus* (GBS), was first described in the late XIX^th^ century in veterinary medicine as a pathogen causing bovine mastitis (Nocard and Mollereau, 1887). In humans this bacterium emerged in the 1960s and represents currently the leading cause of neonatal bacterial infections in developed countries (Eickhoff et al., 1964; Mayon-White, 1985; Gibbs et al., 2004; Da Cunha et al., 2014). *S. agalactiae* is also considered since the 1990s as an emerging pathogen in elderly subjects with underlying conditions (Farley et al., 1993; Skoff et al., 2009). Moreover, for 10–30% of the healthy human population, this bacterium belongs to the commensal microbiota that colonizes the gastrointestinal and genitourinary tracts (Hansen et al., 2004; van der Mee-Marquet et al., 2008). *S. agalactiae* isolates were initially discriminated on the basis of the variability of capsular polysaccharides, distinguishing 10 different serotypes by agglutination, and more recently by a molecular approach (Poyart et al., 2007; Imperi et al., 2010). Among various molecular typing methods multilocus sequence typing (MLST; Jones et al., 2003), and more recently multiple locus variable number of tandem repeats analysis (Haguenoer et al., 2011) were subsequently developed to determine the genetic relationship among isolates of *S. agalactiae* and to define genogroups associated with peculiar clinical issues. MLST, the current reference method for *S. agalactiae* genotyping, is able to distinguish many sequence types (STs), and methods such as eburst (Feil et al., 2004) are able to cluster close STs to define clonal complexes (CC) reflecting the phylogenetic structure of the *S. agalactiae* population. Defined phylogenetic lineages are associated with specific pathogenicity. In particular, CC-17 constitutes a homogeneous group specifically adapted to humans and composed mainly of isolates implicated in the majority of invasive infections in neonates (Poyart et al., 2008) and shows a low rate of recombination (Da Cunha et al., 2014). The others major CC implicated in human infections and/or colonization are CC-1, CC-10, CC-19, and CC-23 (Bohnsack et al., 2008). An analysis of sequenced strains representing the overall species diversity revealed a mosaic organization, with a core genome containing all the ubiquitous genes, and a dispensable genome consisting of partially shared and strain-specific genes organized in genomic islands (Tettelin et al., 2005). These genomic islands are formed by integrative and conjugative elements (ICEs) and prophages (Brochet et al., 2008), suggesting that horizontal gene transfer plays an important role in genome diversification and in the emergence of virulent clones within the species. Indeed, the prophage DNA content, accounting for up to 10% of the dispensable genome, is specific to each intraspecies lineage, highlighting a key role for lysogeny on the evolution of the genetic heritage of bacteria (Domelier et al., 2009; Salloum et al., 2011). However, bacteriophages (phages) exert a constant selective pressure on their bacterial hosts, which in order to survive in this challenging environment have devised various resistance strategies that viruses are trying to escape (Labrie et al., 2010). Thus, in most environments, phages, and bacteria are involved in continuous cycles of co-evolution, in which the emergence of resistant bacteria helps to preserve the bacterial lineage, while the rapid emergence of counter-resistant phages again threatens it. Thus, the investigation of bacterial resistance mechanisms against viruses is critical for the understanding of host–pathogen co-evolution (England and Whitaker, 2013).

Clustered regularly interspaced short palindromic repeats (CRISPR) and CRISPR-associated proteins (Cas) form the CRISPR-Cas system which provides adaptive immunity against invading genetic elements, mainly viruses, ICE, and plasmids, in many bacteria and most archaea (Barrangou et al., 2007; Horvath and Barrangou, 2010; Lopez-Sanchez et al., 2012; Barrangou and Marraffini, 2014; Jiang and Doudna, 2015). The immunity mediated by the CRISPR-Cas system requires the incorporation of DNA fragments from foreign genetic elements into CRISPR arrays, that are subsequently transcribed and processed into small interfering RNAs that guide nucleases (Cas proteins) for targeting cognate genomes in a sequence-specific manner, according to a mechanism which reminds the mechanism of eukaryotic RNA interference (RNAi). CRISPR arrays constitute a peculiar family of DNA repeats, first described by Ishino et al. (1987), and usually constituted of multiple, non-contiguous DNA repeats interspaced by unique sequences of constant length (21–72 bp), named spacers. Most CRISPR arrays are flanked on one side by an AT-rich sequence called leader containing the transcription promoter. The specificity of this DNA-encoded immunity is provided by the spacers sequences that correspond in most cases to segments of captured viral and plasmid sequences (Bolotin et al., 2005; Mojica et al., 2005; Pourcel et al., 2005; Horvath et al., 2008). However, a minority of spacers have been shown to match bacterial chromosome sequences (Stern et al., 2010; Lopez-Sanchez et al., 2012), and their function remains unclear. For some species, it has been demonstrated that such spacers can play a different role than immunity (Louwen et al., 2013). Despite the large diversity of repeats observed across microbial species (Grissa et al., 2007), these sequences, typically 23–47 base pairs (bp), have common properties. Repeats are highly conserved within a given CRISPR array, although limited sequence divergence can be observed, notably for the terminal (opposite to the leader end) repeat (Barrangou et al., 2007; Horvath et al., 2008). Moreover, most repeats are characterized by their partially palindromic nature that allows them to form highly conserved secondary structures (Kunin et al., 2007). Quantitatively, the number of repeat-spacer units per array varies but remains below 50 units in most cases, far behind the current record of 588 units observed in *Haliangium ochraceum* (Ivanova et al., 2010). Microorganisms may contain several distinct CRISPR loci, one or two most frequently, typically located on the chromosome (Mojica et al., 2005; Grissa et al., 2007). CRISPR arrays are in most cases adjacent to *cas* genes that encode a set of functionally very diverse proteins. These proteins carry functional domains typical of helicase, polymerase, nuclease, and polynucleotide-binding proteins (Makarova et al., 2011) and are essential to CRISPR-Cas system activity (Barrangou et al., 2007; Brouns et al., 2008; Szczepankowska, 2012). *cas* genes and the proteins they encode are diverse but usually highly conserved within a given CRISPR-Cas type, and thus represent an important criterion of CRISPR-Cas system classification. CRISPR-Cas systems have been classified into three types (I–III) and a dozen of subtypes based on differences in repeat sequences, Cas protein sequences, and architecture of *cas* operons (Makarova et al., 2011). Universal genes *cas1* and *cas2*, present in all three types, constitute the core of this classification whereas Cas3 nuclease-helicase, Cas9 nuclease, and Cas10 represent the signature proteins for types I, II, and III, respectively. Phylogenetically, Types I and III found both in bacteria and archaea are related, whereas Type II systems, solely present in bacteria, are distinct (Makarova et al., 2013). CRISPR-Cas systems function following three stages (van der Oost et al., 2014), based on the properties of Cas proteins. The stage called adaptation is characterized by the integration of a new spacer into the CRISPR array, which requires Cas1 and Cas2 proteins (Yosef et al., 2012). New invader-derived spacers are integrated into the CRISPR array in a polarized manner at the leader end, accompanied by the duplication of the leader-end (LE) repeat creating a new repeat-spacer unit (Barrangou et al., 2007). Thus, the CRISPR array, when considering both content and sequential order of the spacers, provides a chronological record of past immune conflicts with foreign nucleic acids. The selection of spacer precursors (called proto-spacers; Deveau et al., 2008) from the intruding genetic elements appears to be determined by the recognition of a short flanking sequence (2–5 nt) called protospacer adjacent motif (PAM), which might be different for each given CRISPR-Cas system (Mojica et al., 2009; Paez-Espino et al., 2013). During another stage called expression, a long primary transcript called pre-crRNA is generated from the CRISPR array (from the transcription promoter embedded within the leader sequence) and subsequently cleaved within each repeat sequence by Cas6 nuclease homologs (type I and III systems) or by RNase III (type II systems), producing short interfering RNAs called crRNAs. In the third stage called interference, crRNAs associated with Cas proteins guide Cas nucleases for specific cleavage of the target virus or plasmid sequences.

The adaptive immune system CRISPR-Cas is an acquired defense mechanism, vertically transmitted. The polymorphism and highly evolving nature of CRISPR arrays, in conjunction with their ability to acquire novel spacers in a polarized manner, make them attractive epidemiological markers for genotyping, and phylogenetic analysis of microbial populations. CRISPR array diversity, as a result of both spacer gain (via polarized spacer acquisition) and loss (via internal deletion by homologous recombination between two direct repeats) or error during duplication can be leveraged for genotyping and phylogenetic analysis of medical interest bacteria (Shariat and Dudley, 2014). While LE spacers differentiate closely related strains separated by small evolutionary time scales, CRISPR spacer content also provides valuable information about the common origin of strains when considering the conservation of ancestral spacers located at the leader-distal end, called trailer end (TE). An early application of CRISPR spacer diversity was developed long before the elucidation of CRISPR-Cas functional role, for typing *Mycobacterium tuberculosis* isolates by a hybridization method called spoligotyping (Groenen et al., 1993). Since then, CRISPR array diversity has been successfully used for the genotyping of numerous bacterial species including *Yersinia pestis*, *Corynebacterium diphtheriae*, *Pseudomonas aeruginosa*, *Streptococcus pyogenes*, *Streptococcus thermophilus*, *Campylobacter jejuni*, and *Salmonella enterica* (Horvath et al., 2013). The investigation of intra-species polymorphism at CRISPR loci represents a molecular tool highly promising for the genotyping of many bacteria and the understanding of their evolution. However, it is important to keep in mind that the potential typing scheme varies from one CRISPR locus to another. Indeed, each CRISPR locus has its own specificity that conditions its polymorphism: activity (especially for novel spacer acquisition), distribution and occurrence within the bacterial species, and its propensity to spread by horizontal gene transfer. Epidemiologic potential must be assessed for each bacterial species and each CRISPR locus (Horvath et al., 2013).

Two CRISPR-Cas systems have been identified in *S. agalactiae*, a type II-A system associated with the CRISPR1 locus, and a Type I-C system associated with the CRISPR2 locus. Lopez-Sanchez highlighted the ubiquitous, highly polymorphic, and functional nature of CRISPR1, whereas CRISPR2, only found in ~20% of the strains, showed a low degree of diversity, suggesting little or no activity of the corresponding CRISPR-Cas system (Lopez-Sanchez et al., 2012). This study reported the use of CRISPR1 spacer content for genotyping *S. agalactiae* and, based on similarities between spacers and known sequences, suggested a role for the CRISPR1-Cas system in the regulation of the species’ mobilome.

Our work is a continuation of this previous study and focuses on the analysis of the structure and diversity of CRISPR1 in *S. agalactiae*. In addition to the examination of CRISPR1 spacers, all elements of CRISPR1 (spacers, repeats, and flanking regions) were integrated in our analysis. The aim of our study was to apprehend globally the activity and the role of CRISPR1 in order to assess the pertinence of its use as epidemiological marker and its involvement in the evolutionary dynamics of the *S. agalactiae* species. Our work also differs by the choice of the *in silico* analysis method.

## Materials and Methods

### Bacterial Strains

Our collection consists of 123 epidemiologically unrelated *S. agalactiae* strains of human origin collected in various regions of France during previous epidemiological studies (Domelier et al., 2008; **Table 1**). These strains were selected on the basis of previously established characteristics as representative of the diversity of strains isolated in humans. Fifty-seven of them were carriage strains isolated from samples of various origins (digestive, urogenital, cutaneous, oropharyngeal) in asymptomatic adult patients. These strains, involved in the colonization of the commensal flora, were each identified by a unique number preceded by the letter “C.” Sixty-six of the 123 *S. agalactiae* strains were invasive strains isolated in newborns (*n* = 37) and adults (*n* = 29) from normally sterile samples such as blood cultures (newborns: *n* = 9, adults: *n* = 26) and cerebrospinal fluid (CSF; newborns: *n* = 28, adults: *n* = 3). These strains responsible for invasive diseases were each identified by a unique number preceded by the letter “S.” All strains have been characterized phenotypically and genotypically during previous studies and their capsular serotype, ST, and prophage DNA content are known (Salloum et al., 2010, 2011). Moreover three reference strains of *S. agalactiae* (NEM316, 2603V/R, and A909) which genome has been sequenced and published (Glaser et al., 2002; Tettelin et al., 2005) were included into our study. The distribution of strains according to their origins, their capsular serotype, and ST is presented in **Table 1**.

**Table 1:** Distribution of the 123 *Streptococcus agalactiae* strains studied and the three reference strains (NEM316, A909, 2603V/R), as a function of capsular serotype and origin, within MLST clonal complexes (CC).

Clonal Complex (No. of strains)	Sequence Type (ST) (No. of strains)	Capsular serotype (No. of strains)	No. of invasive strains				No. of colonizing strains
			NN		Ad		
			Blood culture	CSF	Blood culture	CSF	
**CC-1** (26)	ST-1 (16) ST-2 (5) ST-173 (1) ST-196 (3) ST-370 (1)	V (13) II (2) NT (1) Ia (1) Ib (1) II (1) NT (2) V (1) Ib (1) IV (2) V (1)	– – – – –	1 – – – –	6 – 1 – –	– – – – –	9 5 – 3 1
			–	**1**	**7**	–	**18**
**CC-10** (25)	ST-5 (1) ST-6 (1) ST-7 (1) ST-8 (8) ST-10 (5) ST-12 (4) ST-41 (2) ST-255 (1) ST-390 (1)	Ib (1) Ib (1) NT (1) Ib (7) NT (1) Ib (1) IV (1) V (1) NT (2) Ib (3) III (1) Ib (3) III (1) Ib (1) Ib (1)	– – – – – – – – –	– 1 – 1 – – – – –	– – – 1 2 3 – 1 –	– – – – – – – – –	1 – 1 6 3 1 2 – 1
				**2**	**7**	–	**15**
Strain A909	ST-7	Ia					
**CC-17** (30)	ST-17 (30)	III (30)	**6**	**18**	**1**	–	**5**
**CC-19** (14)	ST-19 (10) ST-28 (1) SR-386 (1) ST-389 (1)	III (9) NT (1) V (1) II (1) III (1)	– – – –	3 – – –	3 – 1 –	1 – – –	3 1 – 1
			–	**3**	**4**	**1**	**5**
Strain 2603V/R	ST-110	V					
**CC-23** (26)	ST-23 (17) ST-144 (1) ST-220 (2) ST-223 (1) ST-305 (1) ST-385 (1) ST-391 (1) ST-481 (1)	Ia (15) III (2) Ia (1) Ia (2) Ia (1) Ia (1) Ia (1) III (1) III (1)	2 – – – – – – 1	3 – – – – – – –	5 – – – – 1 – –	2 – – – – – – –	5 1 2 1 1 – 1 –
			**3**	**3**	**6**	**2**	**11**
Strain NEM316	ST-23	III					
**Singletons** (5)	ST-388 (2) ST-4 (1) ST-24 (1) ST-130 (1)	V (2) Ia (1) Ia (1) V (1)	– – – –	– – 1 –	– 1 – –	– – – –	2 – – 1
			–	**1**	**1**	–	**3**
**Total**			**9**	**28**	**26**	**3**	**57**

*NN, neonates; Ad, adults; CSF, cerebrospinal fluid.*

*NT, non-typable.*

### DNA Extraction

*Streptococcus agalactiae* strains were stored at −80°C in a medium containing glycerol at a final concentration of 20% (vol/vol), and were grown in trypticase soy agar supplemented with 5% horse blood (TSH agar, Biomerieux) for 24 h at 37°C in ambient air. Genomic DNA from each strain was extracted following enzymatic lysis with mutanolysin (Sigma–Aldrich). To do so, bacterial suspension of OD600 0.3 was prepared in a volume of 500 μl of water for extraction (Argene, Biomerieux) containing 50 U of mutanolysin. Lysis was achieved by suspension incubation for 1 h at 56°C followed by 10 min at 100°C. Lysates were centrifuged at 15000 × *g* for 3 min, and the supernatant containing DNA was collected. The concentration of the obtained DNA was estimated by spectrophotometry for each strain.

### CRISPR1 Locus Amplification

Polymerase chain reactions (PCRs) for CRISPR1 locus amplification were performed in a T3000 Thermocycler (Biometra) with Q5 High-Fidelity DNA polymerase^®^ (New England Biolabs) and the oligonucleotide pair CRISPR1-PCRF and CRISPR1-PCRR targeting CRISPR1 flanking regions (Lopez-Sanchez et al., 2012). PCR amplifications were performed in a total volume of 25 μl containing 50 ng of template DNA, 0,5 μM forward and reverse primers, 0,2 mM deoxynucleoside triphosphates (dNTPs), 2 mM MgCl_2_, 0,02 U/μl of Q5 High-Fidelity DNA polymerase^®^ and 1x buffer. The cycling conditions were as follows: 5 min for denaturation at 98°C, followed by 40 cycles of 30 s at 98°C for denaturation, 30 s at 56°C for annealing, and 120 s at 72°C for extension, followed by 10 min at 72°C for final extension. Positive PCR amplification was verified by electrophoretic migration into a 1% agarose gel. PCR product size was estimated by comparison with the molecular weight size marker “ExactLadder DNAPreMix 2log^®^” (Ozyme).

### Amplicon Purification and CRISPR1 Locus Sequencing

All PCR products were purified using the Centrifugal Filter Units^®^ (Millipore Corporation) in accordance with the manufacturer’s recommendations. The purified products were sequenced with BigDyeTerminator^®^ Mix v3.1 (Applied Biosystems) and a pair of internal sequencing primers previously described (Lopez-Sanchez et al., 2012) on a Hitachi 3130xl Genetic Analyzer (Applied Biosystems). For CRISPR regions exceeding 1,3 kb, a primer-walking strategy using primers designed within spacer sequences was performed to complete sequencing of the PCR products (Supplementary Table S1).

### *In silico* Analysis of CRISPR Loci

The obtained DNA sequences were analyzed, edited, and assembled using the softwares 4Peaks v1.7.1 (Mekentosj) and ApE v2.0.47 (Biologylabs). For each sequence, spacers, repeats, and flanking regions were determined and collected in a database by using different tools specifically dedicated to the analysis of CRISPR loci. Initially, CRISPRfinder and CRISPRtionary applications^1^ (Grissa et al., 2007) were used to retrieve and find the CRISPR1 locus structure, and to generate dictionary of spacers, respectively. The use of the CRISPRtionary tool by Lopez-Sanchez allowed the establishment of a dictionary of 949 spacers, numbered from 1 to 949 (Lopez-Sanchez et al., 2012). New spacers identified in this study expanded this dictionary. In a second step, CRISPR1 array structure was determined anew using a macro-enabled Excel tool named *CRISPR database II* (P. Horvath, DuPont) that comprises different programs for the identification and extraction of CRISPR features in nucleotide sequences, and for subsequently establishing a graphic representation of spacer diversity.

The similarity of each spacer sequence, repeat sequence and flanking regions to the microbial genome database in GenBank^2^ was analyzed by BLASTn (Altschul et al., 1997) using an *E*-value cut-off of 0.1. Only matches to elements located outside the *S. agalactiae* CRISPR1 array were considered as legitimate hits. All matches with a bit score above 40.0 and a query cover above 80% (corresponding to 100% identity over at least 24 bp) were retained. If multiple annotations were proposed, only the top hit annotation was considered for categorization.

### Statistical Analysis

The distribution of the number of spacers with respect to CC was compared using the non-parametric Kruskal–Wallis test. The test was considered significant for *p* < 0.05.

## Results

### Analysis of the CRISPR1 Locus in *S. agalactiae*

**FIGURE 1:**
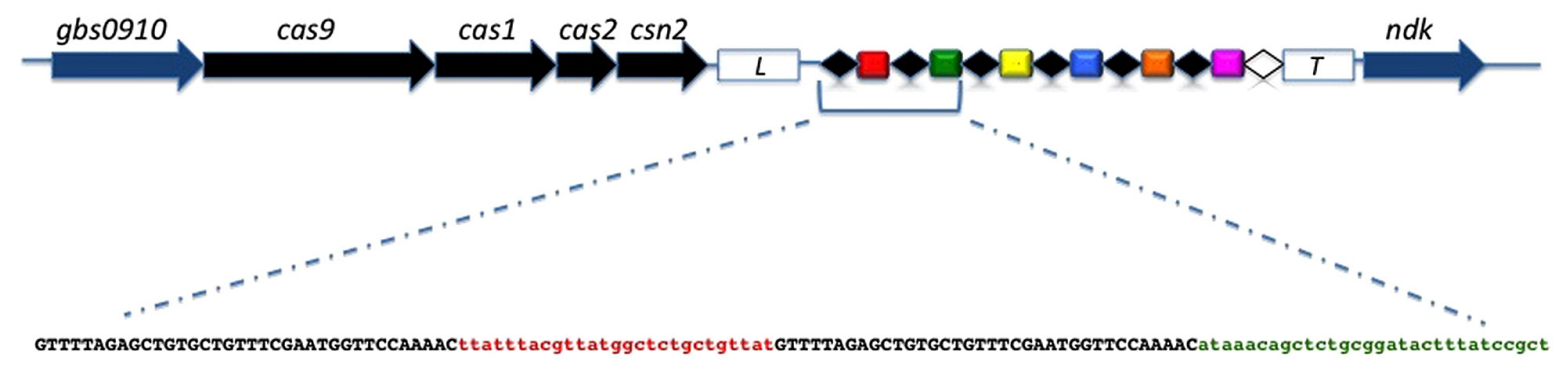
**Genetic organization of the CRISPR1-*cas* locus in *Streptococcus agalactiae*.** *cas* genes and core genes are shown as black arrows and blue arrows, respectively. The leader sequence is located between the *cas* gene cluster and the CRISPR array (white box; L) while the trailer sequence is located downstream of the array (white box; T). The direct repeats (DR) are shown as black diamonds and the terminal repeat, which differs from the consensus DR, is shown as a white diamond. Spacers are shown as colored rectangles and unique spacers are represented by unique colors. Below the CRISPR array, the sequence of the first two repeat-spacer units is shown with the DRs in black characters and the spacers in color characters.

Polymerase chain reaction amplification of the CRISPR1 locus was positive for all the strains, confirming the ubiquitous nature and conserved structure of the type II-A CRISPR1 locus in *S. agalactiae* (Lopez-Sanchez et al., 2012). For each strain a single DNA amplicon was obtained with a size varying between 1,100, and 2,700 bp, corresponding to a CRISPR1 array size between 200 and 1,800 bp. The complete sequence of the CRISPR1 locus could be generated for each strain, which allowed the sequence analysis of repeats, spacers, leader, and trailer. An overview of the CRISPR1-*cas* locus in *S. agalactiae* is presented in **Figure 1**.

### Analysis of CRISPR Repeats

The typical repeat is conventionally defined as the most frequent repeat within a CRISPR array. Usually, each CRISPR array is defined by the sequence of the typical repeat which is generally highly conserved throughout the array. Among the 126 strains 1,837 repeats were identified, including 126 terminal repeats that delimit the distal end of the CRISPR1 array. Analysis of repeats highlighted a highly conserved typical repeat, present in all strains and representing 92% of the 1,837 repeats identified, and 98.5% of the internal repeats (**Table 2**). The typical repeat is a nearly perfect 36-bp palindrome (5′-GTTTTAGAGCTGTGCTGTTTCGAATGGTTCCAAAAC-3′) previously described by Lopez-Sanchez et al. (2012). We analyzed the typical repeat sequence and investigated similarity to bacterial sequences using BLASTn and CRISPRdb (Grissa et al., 2007). The typical repeat showed sequence similarity, according to predefined criteria (see Material and Methods), to others typical repeat sequences present in species of the *Streptococcus* genus. CRISPR1 typical repeat sequence in *S. agalactiae* perfectly matches CRISPR repeats found in *S. anginosus*, *S. infantarius*, *S. lutetiensis*, and *S. gallolyticus* ssp. *gallolyticus*; diverging by only one nucleotide with CRISPR repeats in *S. thermophilus* and *S. mutans*; and by three nucleotides with CRISPR repeats in *S. pyogenes* and *S. equi* ssp. *zooepidemicus*.

**Table 2:** Inventory and distribution of CRISPR1 repeat sequences among *S. agalactiae* CC.

Type	Repeat sequence (5′- 3′)	Frequency (%)	Clonal complex or ST(No. of strains)
**Internal repeat**		
Typical repeat	GTTTTAGAGCTGTGCTGTTTCGAATGGTTCCAAAAC	98.5	CC-1 (26)CC-10 (25)CC-17 (30)CC-19 (14)CC-23 (26)ST-4 (1)ST-388 (2)ST-130 (1)ST-24 (1)
Repeat variants	GTTTTAGTGCTGTGCTGTTTCGAATGGTTCCAAAAC	0.1	CC-17 (2)*
	GTTTTAGAGCTGTGCTATTTCGAATGGTTCCAAAAC	0.1	CC-23 (1)*
	GTTTTAGAGCTGTGTTGTTTCGAATGGTTCCAAAAC	1.2	CC-23 (4)*
	GTTTTAAAGCTGTGCTGTTTCGAATGGTTCCAAAAC	0.1	CC-19 (1)*
**Terminal repeat**			
Typical terminal repeat	GTTTTAGAGCTGTGCTGTTATTATGCTAGGACATCA	52	CC-1 (26)CC-10 (25)CC-19 (14)ST-4 (1)
Terminal repeat variants	GTTTTAGAGCTGTGCGGTTATTATGCTAGGGCACCG	25	CC-17 (30)ST-130 (1)
	GTTTTAAAGCTGTGCTGTTATTATGCTAGGGCACCA	21.5	CC-23 (26)ST-24 (1)
	GTTTTAGAGCTGTGCTGTTATTATGCTAGGGCACCA	1.5	ST-388 (2)

Nucleotide polymorphisms as compared to the typical CRISPR repeat sequence are indicated in red.

*All CRISPR1 arrays with at least a repeat variant contain also the typical repeat.

Clustered regularly interspaced short palindromic repeats1 sequence comparisons showed that although the repeat sequence is usually highly conserved throughout the array, polymorphisms can be observed. Rare polymorphisms were observed in the repeat sequence, leading to four variants (or atypical repeats) that carry single-nucleotide polymorphisms and represent 25 of the 1,837 repeats identified (1.5%; **Table 2**). As observed previously in other organisms, the main repeat polymorphism is located at the distal end of each CRISPR array, in the terminal repeat. For all strains, the terminal repeat showed sequence degeneracy predominantly at the 3′ end of the repeat, marking the boundary of the CRISPR1 array. We found four different terminals repeats of very similar sequence, showing 50–70% identities with the typical internal repeat. Interestingly, the distribution of the strains according to the sequence of their terminal repeat is almost perfectly correlated with MLST typing results. Strains of CC CC-17 and CC-23, and of ST-388 (CC26) are characterized by the presence of a specific terminal repeat, whereas CRISPR1 arrays of strains grouped into CC-1, CC-19, and CC-10 have the same terminal repeat.

### Analysis of CRISPR1 Flanking Regions

The leader sequence, directly adjacent to the first CRISPR1 repeat, was identified in all the 126 *S. agalactiae* strains. As described previously in other organisms, the CRISPR1 leader is an A/T-rich (63%) sequence, which is highly conserved within the species. However, the comparison of leader sequences showed three types of variations of this sequence. The most common variation was only observed in CC-10 strains (13 out of 24), where one nucleotide is missing at the 3′end of the leader sequence. Other variations, observed in one CC-1 strain, one CC-19 strain, and one ST-130 strain, were an addition of three nucleotides or one nucleotide, and a nucleotide substitution at the 3′end of the leader sequence, respectively (Supplementary Table S2). The leader sequence showed no similarity (using BLASTn and CRISPRdb) to other bacterial sequences, suggesting that the leader sequence is specific to *S. agalactiae*.

The sequence of the flanking region located downstream of CRISPR1 (trailer end) was identified and strictly conserved in all (126) *S. agalactiae* strains. This region is a non-coding, 540-bp long sequence located between CRISPR1 and the *ndk* gene encoding a nucleoside diphosphate kinase. This sequence also showed no similarity to other bacterial sequences, suggesting again a CRISPR1 specificity.

### Analysis of CRISPR1 Spacers

Across the 126 *S. agalactiae* strains analyzed, we identified 1,714 spacers, of which 450 (26%) were unique. Among these, 258 spacers were previously described by Lopez-Sanchez et al. (2012), and 192 spacers corresponded to new spacers that were incrementally numbered following nomenclature.

#### CRISPR1 Spacer Polymorphism

**FIGURE 2:**
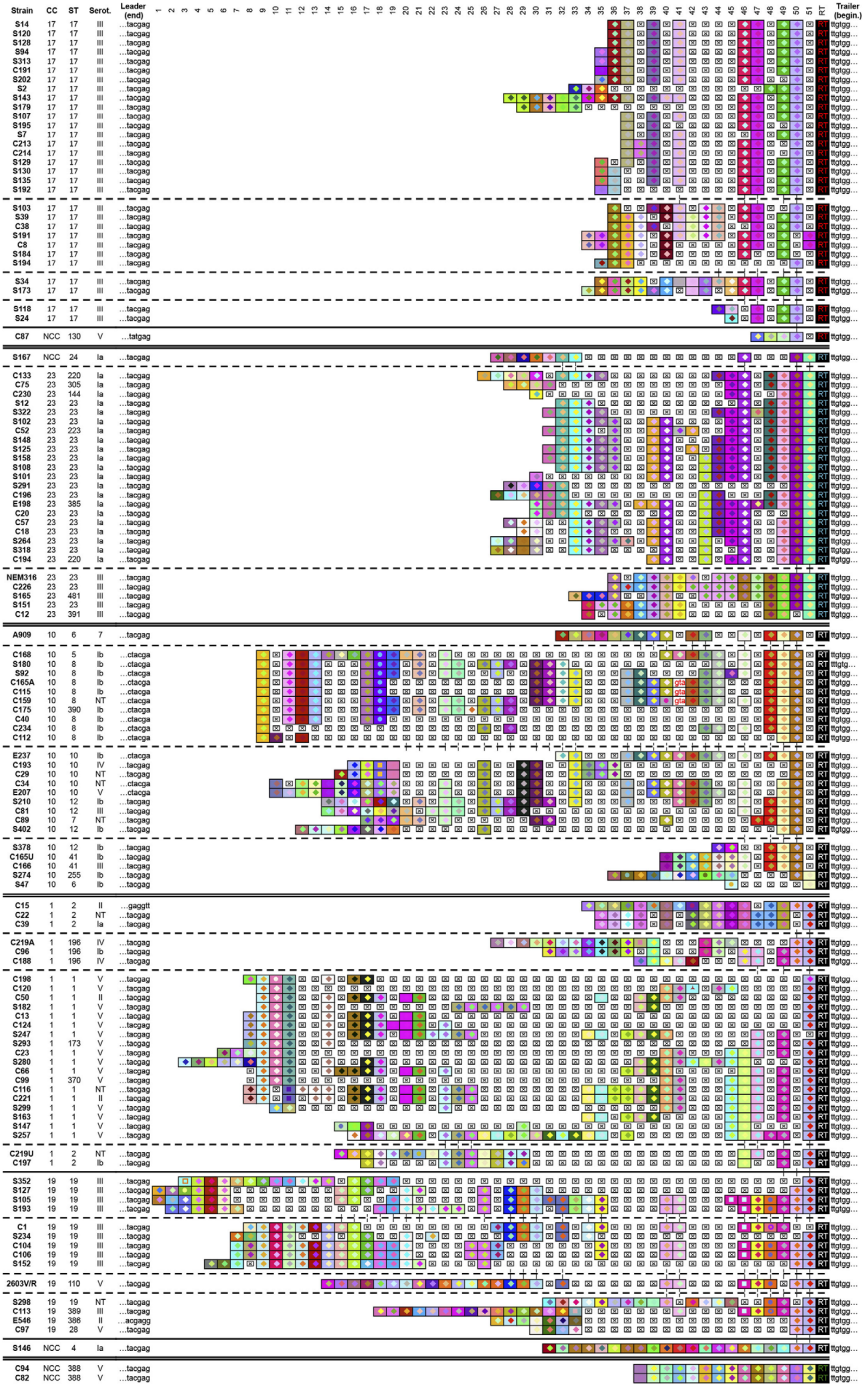
**Graphic representation of CRISPR1 loci for 126 *S. agalactiae* strains**. Internal repeats are not included; only terminal repeats (RT), the leader and trailer end sequences (last six nucleotides and first six nucleotide, respectively) and spacers are represented. Each spacer is represented by a combination of one select character in a particular front color, on a particular background color. The color combination allows unique representation of a particular spacer, whereby squares with similar color schemes (combination of character color and background color) represent identical spacers, whereas different color combinations represent distinguishable spacers. Deleted spacers are represented by crossed squares. Strain names, clonal complexes (CC), sequence type (ST), and capsular serotype are given on the left. NCC indicates strains that do not belong to CC according to MLST. NT indicates strains that are not discriminated on the basis of the variability of capsular polysaccharides. Strains were arranged according to the CRISPR1 content. A double line separates CRISPR1 groups. Broken lines separate distinct subgroups in CRISPR1 groups and a continuous line separates NCC strains in CRISPR1 groups.

Investigation of CRISPR1 spacer diversity across 126 *S. agalactiae* strains identified 115 unique spacer arrangements (93%), indicating that the high polymorphism of CRISPR1 spacers in both sequence and number provides a higher strain discrimination capacity than other subtyping techniques such as MLST, which separates these same strains into only 31 distinctive STs, as previously described (Lopez-Sanchez et al., 2012). Graphic representation of spacers across the CRISPR1 array for the 126 *S. agalactiae* strains is presented in **Figure 2**.

Spacer polymorphisms were relatively rarely due to spacer size differences. The typical spacer size, 30 bp, is highly conserved (397/418 = 95%), with remaining spacers ranging in size between 28 and 33 bp. A noteworthy exception was a 58-bp spacer of which the first 30 bp and the last 28 bp were identical to two contiguous known spacers, suggesting that this atypical spacer resulted from the deletion of the repeat located in-between.

The number of spacers in each array displays important variations across the different *S. agalactiae* strains examined, with an average number of 13.6 spacers per array, and minimum and maximum numbers of 2 (S47 from CC-10) and 29 spacers (S193 from CC-19), respectively.

In several cases, variations between strains resulted from CRISPR1 locus microevolution, leading to mere deletion, addition, or duplication of one or more repeat-spacer units. There is a pronounced disparity in these phenomena of microevolution among *S. agalactiae* strains since some strains presented microvariations involving a single repeat-spacer unit, whereas in others large segments appeared deleted or duplicated.

We also occasionally observed discrete changes in sequence of CRISPR1 spacers, revealing the presence of variant spacers. Among the 450 distinctive spacers, 25 (6%) have a nucleotide sequence varying of one or two nucleotides compared to the sequence of previously identified spacers. The variations are mainly located at 5′- and 3′-end nucleotides of the spacers and are characterized by the gain or loss of a nucleotide at either end. Rarely variant spacers differed by single nucleotide polymorphism in the middle of the spacer. These variant spacers may have a common origin and variations could be due to point mutations.

#### Analysis of CRISPR1 Spacer Sequences

We investigated the sequence similarity of these 167 new spacers (the 25 variant spacers were not included in this analysis) to phage, plasmid, and bacterial sequences. Among 62 spacers (37%) showing matches, 40 (64%) showed similarity to viral sequences, while 2 (3%) were similar to plasmid sequences, and 20 (33%) matched CRISPR-unrelated chromosomal sequences in *S. agalactiae*, *S. dysgalactiae*, or *S. parauberis*. These potential chromosomal target sequences were found in only a few isolates and could correspond to mobile genetic elements (MGEs, i.e., ICEs or phages) inserted in bacterial genomes. Overall, taking into account all 207 matching spacers (out of 450; 46%), the large majority matches sequences present in one or several of the complete *S. agalactiae* genomes, and corresponds most often to MGEs inserted in these genomes (LambdaSa3, LambdaSa1, and Phi3396 prophages, and Tn*GBS2*). Spacers not matching *S. agalactiae* MGEs matched either MGEs inserted in other *Streptococcus* genomes (for instance prophages JX01 and LYG09), or more rarely the core genome of *S. agalactiae*. This distribution is consistent with that previously reported by Lopez-Sanchez et al. (2012).

### CRISPR1-Based Clustering

*Streptococcus agalactiae* CRISPR1-based genotyping presents a high discriminatory potential, mainly due to spacer polymorphism and the presence of distinctive terminal repeat variants.

We highlighted above the correlation between strain distribution according to the sequence of their CRISPR1 terminal repeat and their clustering based on MLST (**Table 2**). Moreover, a spacer conservation gradient across the CRISPR1 array was observed. In general, identical spacers between strains occur more frequently at the trailer end of the array and thus appear relatively stable (“oldest” spacers). In contrast, spacers located close to the leader showed more variability and are frequently unique or present in just a few strains (novel spacers). This observation is in agreement with the mechanism of insertion of new spacers at the leader end, and with a reduced propensity of trailer-end spacers (especially the last one) to be lost through deletion events, since the last repeat is different in sequence (**Table 2**; Horvath et al., 2008). The identification of a common set of consecutive spacers at the trailer end of the array in distinct strains implies the existence of a relatively recent common ancestor for these strains.

Accordingly, in a first step the analysis of spacers located at the trailer end of the array, along with the terminal repeat allowed the clustering of all 126 *S. agalactiae* strains into four groups (**Figure 2**). In a second step, the analysis of internal (more recently acquired, i.e., closer to the LE) spacer composition separated each group into various subgroups. Comparison between the distribution of strains across CRISPR1 groups and their MLST classification showed an evident correlation. The CRISPR1 group affiliation, defined by the trailer-end structure of the array, is closely related to MLST-based CC.

Four trailer-end spacers and a specific terminal repeat define CC-17, two trailer-end spacers and another specific terminal repeat define CC-23, two trailer-end spacers define CC-10, and three trailer-end spacers define CC-1 and CC-19 that belong to the same CRISPR1 group. However, strains related to these two latter CCs could be mostly individualized into two subgroups on the basis of internal spacers composition. Strains of CC-10, CC-1, and CC-19 have the same CRISPR1 terminal repeat, which is the typical terminal repeat. Note that the last CRISPR1 group is a singleton and contains two ST-388 strains characterized by an identical spacer composition and a specific terminal repeat. As previously described by Lopez-Sanchez et al. (2012), in some CRISPR1 groups a concordance between subgroups and the results of capsular serotyping or MLST was observed. For example in CC-23, the internal spacer composition strictly separates serotype III and Ia strains into two subgroups (**Figure 2**). Similarly, strains from CC-1 are split into four subgroups, distinguishing ST-196 from the other major STs (ST-2 and ST-1). In some cases CRISPR1 typing was able to successfully discriminate strains that were considered indistinguishable by MLST. The homogeneous phylogenetic lineage composed exclusively of ST-17 strains (capsular serotype III) appears separated into distinctive subgroups. It is important to note that three strains (out of 5) that do not belong to CC according to MLST turn out to be related to CC-17 (C87: ST-130), CC-23 (S167: ST-24), and CC-1 or CC-19 (S146: ST-4) on the basis of CRISPR1 composition. Among the 126 *S. agalactiae* strains, only ST-388, constituting a distinct CRISPR1 group, appears isolated in the CRISPR1-based strain distribution.

In-depth analysis of the spacer composition reveals a marked difference in diversity among subgroups. For some subgroups, the CRISPR1 spacers close to the leader are relatively preserved and common to other strains within the same subgroup, suggesting a reduced ability to further acquire novel spacers. In others subgroups a higher spacer diversity is observed at the leader end, while internal spacers seem to have been deleted *en bloc*, suggesting a possible link between spacer acquisition propensity and loss of relatively ancient spacers.

**FIGURE 3:**
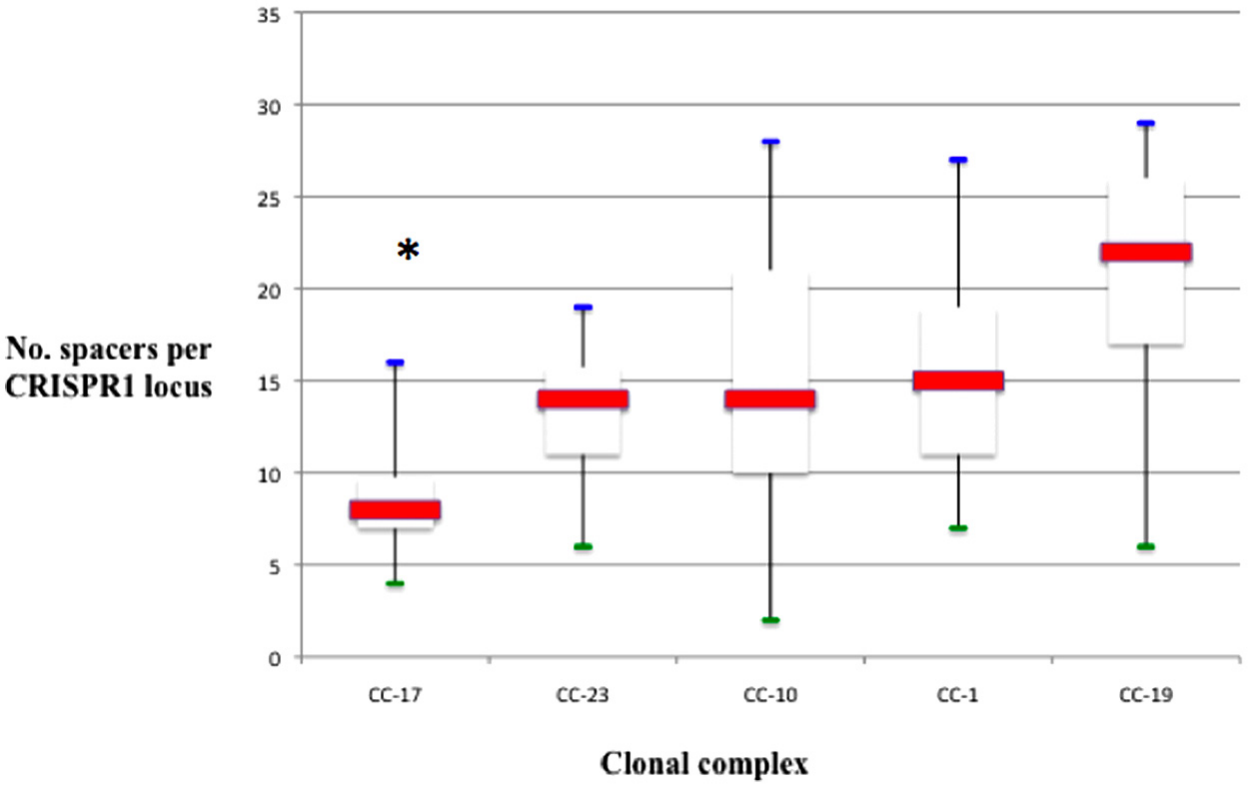
**Number of spacers per locus, represented in box plots, among different CC.** The red line is the median. The lower end and the upper end of the rectangle represent the first and third quartile, respectively. The blue and green lines represent maximum and minimum values, respectively. The number of spacers in locus CRISPR1 was significantly lower for strains belonging to CC-17 than for others CC (*p < 0.001).

Remarkably, the CRISPR1 analysis reveals different levels of polymorphism among CC as defined by MLST. CC-17 showed the lowest diversity, followed by CC-23 and the three CC CC-1, CC-19, and CC-10 (**Figure 2**). The proportion of unique spacer arrangements is greater than 90% for all the CCs, except for CC-17 (80%). Overall, the degree of polymorphism was the lowest for CC-17 in terms of unique spacers, unique spacer arrangements, and average spacer number (**Figure 3**).

## Discussion

Analysis of the structure and content of the CRISPR1 locus in 126 *S. agalactiae* strains confirmed its ubiquitous nature despite a high spacer sequence polymorphism, as described by Lopez-Sanchez et al. (2012). Our results, using a methodology allowing a careful analysis of the flanking regions, repeats, and spacers of the CRISPR1 locus, provide additional evidence that CRISPR1 typing not only correlates strongly with MLST, but also provides deeper discrimination than the current reference method for *S. agalactiae* typing. Variations in CRISPR1 spacer content were already credited to various mechanisms such as acquisition, deletion, and internal duplication of one or several repeat-spacer units. In addition to these alteration causes, we identified the occasional presence of variant spacers likely due to point mutations, and more importantly the systematic presence of a degenerated terminal repeat (and the associated, previously missed upstream spacer) which sequence defines groups that match perfectly MLST-based clustering.

The polymorphism observed in spacer content of a CRISPR array is considered to be an indicator of the functional activity of the locus. The ubiquitous presence of this CRISPR-Cas system in the species, the low level of repeat sequence polymorphism, with the exception of terminal repeats, the absence of insertion sequences, the conservation of flanking regions, and the spacer size homogeneity are other arguments in favor of CRISPR1 activity, as previously described for CRISPR loci in *S. thermophilus* (Horvath et al., 2008). Indeed, the interference effectiveness of *S. agalactiae* A909 CRISPR1-Cas system was demonstrated in laboratory conditions under a selective pressure of TnGBS2, an ICE originating from *S. agalactiae* NEM316 (Lopez-Sanchez et al., 2012).

The tight congruence between MLST and CRISPR1-based genotyping highlights the usefulness of the CRISPR1 locus as an effective epidemiological marker in *S. agalactiae*. CRISPR-based typing methods have been well established for some bacterial species such as *M. tuberculosis* (Lanotte, 2012) and have been extended to many other human pathogens, as illustrated by their application to recent *Salmonella enterica* epidemic episodes (Fabre et al., 2012; Shariat et al., 2013). Most often CRISPR-based typing methods provide discriminatory power and epidemiological concordance that are at least equivalent, if not superior, to the commonly used typing methods (Shariat and Dudley, 2014). However, it is essential to assess, on a case-by-case basis, the characteristics of any given CRISPR locus before its use as an epidemiological marker. Actually not all CRISPR loci are appropriate for molecular subtyping, as illustrated by CRISPR2 in *Escherichia coli* which has been widely disseminated through horizontal transfer, altering the epidemiological concordance and therefore prohibiting its use as typing target (Touchon et al., 2011).

Beyond strain differentiation, high-resolution CRISPR1 typing provides opportunities to improve our perception of *S. agalactiae* population evolution and to explore the species’ genetic diversity. The existence of strong similarity between the typical CRISPR1 repeat sequence and CRISPR repeats in many other streptococcal species such as *S. pyogenes* and *S. anginosus* suggests a wide distribution of this type II-A CRISPR-Cas system within the genus.

Analysis of the *S. agalactiae* genome showed a mosaic organization formed by large chromosomal fragments from different ancestors, suggesting that large DNA exchanges have contributed to genome dynamics in the natural population (Brochet et al., 2009). A model has been proposed for the evolutionary history of this species, in which emergence of CC of clinical importance could be linked to selective sweeps associated with the reduction of genetic diversity (Brochet et al., 2009). The CC-1 and CC-10 strains are characterized by extensive genetic diversity, in contrast to CC-23 and particularly CC-17 strains.

Interestingly, CRISPR1 analysis of the *S. agalactiae* population revealed different degrees of heterogeneity among CC. Indeed, the two CRISPR1 groups containing CC-1/CC-19 and CC-10 strains are characterized by the same terminal repeat and appear more heterogeneous, as evidenced by their separation into many subgroups. Conversely, CRISPR1 groups containing CC-17 and CC-23 strains seem more homogeneous and are characterized by a different, specific terminal repeat. Moreover, they are characterized by a smaller number of spacers, especially for CC-17. CRISPR1 spacer composition and diversity could reflect this evolutionary history in which clones emerging from a heterogeneous population would be distinguished by a more homogeneous CRISPR1 array and the presence of a specific terminal repeat.

The CRISPR1 array characteristics could therefore lead to the development of concrete applications for investigating the transmission mode (horizontal or vertical) of neonatal bacterial infections or the source of a contamination, or for the rapid detection of a particular lineage such as “hypervirulent” ST-17 strains. In fact, CRISPR1 sequence analysis provides a “one-shot” approach for typing, with the benefits of much-reduced time and cost compared to MLST. Invasive infections in elderly adults are characterized for 5% of cases by episodes of recurrent infections associated with the same strain, requiring the search for a deep infection localization (Farley, 2001). In this case, the comparison of two isolates responsible for distinct infectious events by CRISPR1 sequencing could be a valuable alternative to MLST to differentiate recurrent infections from reinfection. For instance, a PCR assay targeting specific CRISPR1 spacers could be developed for this purpose, analogously to the multiplex PCR assay used for the detection of *Salmonella* serovar Typhi and *Salmonella* serovar Paratyphi A (Shariat and Dudley, 2014). In another approach the terminal repeat polymorphisms that are group-specific could be leveraged by high-resolution melt DNA (HRM) to rapidly differentiate alleles, as previously described for *Campylobacter* strains (Price et al., 2007).

Despite the high discriminatory power, reproducibility, portability, and epidemiological concordance of CRISPR1 typing, limitations exist for its use in diagnostic, epidemiologic, and evolutionary analyses of *S. agalactiae* strains. The occasional absence (due to internal deletion) of group- or subgroups-specific spacers prevents or reduces the typing resolution and decreases the congruence with MLST. This limit is especially important to consider for strains of CC-1 and CC-19 that are classified into the same CRISPR1 group. However, our analysis, providing a snapshot of CRISPR1 array content and reflecting its dynamism, suggests that these events minimally impact the effectiveness of CRISPR1 typing.

The selective advantage provided by CRISPR-Cas systems is controversial, some evolutionary models suggesting that their main advantage is resistance against lytic phages (Levin, 2010; Touchon et al., 2011). Paradoxically, similarity searches with *S. agalactiae* spacer sequences against the GenBank database highlighted a large proportion of spacers targeting MGEs widespread among *S. agalactiae* genomes. Accordingly, Lopez-Sanchez proposed that CRISPR1 is selected for at the population level to ensure the coexistence of carrier and non-carrier strains, thus preserving the diversity of the *S. agalactiae* mobilome (Lopez-Sanchez et al., 2012).

## Conclusion

Our work identified new sources of diversity within the CRISPR1 array notably a degenerate terminal repeat delineating a previously missed spacer, and showed the conservation of other structural elements such as the typical repeat. All CRISPR1 features described here are valuable for the epidemiological typing of *S. agalactiae*, providing a superior discriminatory power than MLST. We propose that the content and diversity of the CRISPR1 array reflect the evolutionary process determining population structure, making the sequencing of this locus an attractive tool for phylogenetic studies.

## Conflict of Interest Statement

The authors declare that the research was conducted in the absence of any commercial or financial relationships that could be construed as a potential conflict of interest.

## Footnotes

1 http://crispr.u-psud.fr

2 http://www.ncbi.nlm.nih.gov

## References

[B1] Altschul S. F. Madden T. L. Schäffer A. A. Zhang J. Zhang Z. Miller W. (1997). Gapped BLAST and PSI-BLAST: a new generation of protein database search programs. *Nucleic Acids Res.* 25 3389–3402. 10.1093/nar/25.17.3389 9254694 PMC146917

[B2] Barrangou R. Fremaux C. Deveau H. Richards M. Boyaval P. Moineau S. (2007). CRISPR provides acquired resistance against viruses in prokaryotes. *Science* 315 1709–1712. 10.1126/science.1138140 17379808

[B3] Barrangou R. Marraffini L. A. (2014). CRISPR-Cas systems: prokaryotes upgrade to adaptive immunity. *Mol. Cell* 54 234–244. 10.1016/j.molcel.2014.03.011 24766887 PMC4025954

[B4] Bohnsack J. F. Whiting A. Gottschalk M. Dunn D. M. Weiss R. Azimi P. H. (2008). Population structure of invasive and colonizing strains of *Streptococcus agalactiae* from neonates of six U.S. Academic Centers from 1995 to 1999. *J. Clin. Microbiol.* 46 1285–1291. 10.1128/JCM.02105-07 18287314 PMC2292926

[B5] Bolotin A. Quinquis B. Sorokin A. Ehrlich S. D. (2005). Clustered regularly interspaced short palindrome repeats (CRISPRs) have spacers of extrachromosomal origin. *Microbiology* 151 2551–2561. 10.1099/mic.0.28048-0 16079334

[B6] Brochet M. Couve E. Bercion R. Sire J.-M. Glaser P. (2009). Population structure of human isolates of *Streptococcus agalactiae* from dakar and bangui. *J. Clin. Microbiol.* 47 800–803. 10.1128/JCM.01103-08 19109468 PMC2650903

[B7] Brochet M. Couvé E. Glaser P. Guédon G. Payot S. (2008). Integrative conjugative elements and related elements are major contributors to the genome diversity of *Streptococcus agalactiae*. *J. Bacteriol.* 190 6913–6917. 10.1128/JB.00824-08 18708498 PMC2566197

[B8] Brouns S. J. J. Jore M. M. Lundgren M. Westra E. R. Slijkhuis R. J. H. Snijders A. P. L. (2008). Small CRISPR RNAs guide antiviral defense in prokaryotes. *Science* 321 960–964. 10.1126/science.1159689 18703739 PMC5898235

[B9] Da Cunha V. Davies M. R. Douarre P.-E Rosinski-Chupin I. Margarit I. Spinali S. (2014). *Streptococcus agalactiae* clones infecting humans were selected and fixed through the extensive use of tetracycline. *Nat. Commun.* 5 4544 10.1038/ncomms5544 PMC4538795 25088811

[B10] Deveau H. Barrangou R. Garneau J. E. Labonté J. Fremaux C. Boyaval P. (2008). Phage response to CRISPR-encoded resistance in *Streptococcus thermophilus*. *J. Bacteriol.* 190 1390–1400. 10.1128/JB.01412-07 18065545 PMC2238228

[B11] Domelier A.-S. van der Mee-Marquet N. Arnault L. Mereghetti L. Lanotte P. Rosenau A. (2008). Molecular characterization of erythromycin- resistant *Streptococcus agalactiae* strains. *J. Antimicrob. Chemother.* 62 1227–1233. 10.1093/jac/dkn388 18786936

[B12] Domelier A.-S. van der Mee-Marquet N. Sizaret P.-Y. Héry-Arnaud G. Lartigue M.-F. Mereghetti L. (2009). Molecular characterization and lytic activities of *Streptococcus agalactiae* bacteriophages and determination of lysogenic-strain features. *J. Bacteriol.* 191 4776–4785. 10.1128/JB.00426-09 19465660 PMC2715722

[B13] Eickhoff T. C. Klein J. O. Daly A. K. Ingall D. Finland M. (1964). Neonatal sepsis and other infections due to group B beta-hemolytic Streptococci. *N. Engl. J. Med.* 271 1221–1228. 10.1056/NEJM196412102712401 14234266

[B14] England W. E. Whitaker R. J. (2013). Evolutionary causes and consequences of diversified CRISPR immune profiles in natural populations. *Biochem. Soc. Trans.* 41 1431–1436. 24256233 10.1042/BST20130243

[B15] Fabre L. Zhang J. Guigon G. Le Hello S. Guibert V. Accou-Demartin M. (2012). CRISPR typing and subtyping for improved laboratory surveillance of *Salmonella* infections. *PLoS ONE* 7:e36995 10.1371/journal.pone.0036995 PMC3356390 22623967

[B16] Farley M. M. (2001). Group B streptococcal disease in nonpregnant adults. *Clin. Infect. Dis.* 33 556–561. 10.1086/322696 11462195

[B17] Farley M. M. Harvey R. C. Stull T. Smith J. D. Schuchat A. Wenger J. D. (1993). A population-based assessment of invasive disease due to group B *Streptococcus* in nonpregnant adults. *N. Engl. J. Med.* 328 1807–1811. 10.1056/NEJM199306243282503 8502269

[B18] Feil E. J. Li B. C. Aanensen D. M. Hanage W. P. Spratt B. G. (2004). eBURST: inferring patterns of evolutionary descent among clusters of related bacterial genotypes from multilocus sequence typing data. *J. Bacteriol.* 186 1518–1530. 10.1128/JB.186.5.1518-1530.2004 14973027 PMC344416

[B19] Gibbs R. S. Schrag S. Schuchat A. (2004). Perinatal infections due to group B *Streptococci*. *Obstet. Gynecol.* 104 1062–1076. 10.1097/01.AOG.0000144128.03913.c2 15516403

[B20] Glaser P. Rusniok C. Buchrieser C. Chevalier F. Frangeul L. Msadek T. (2002). Genome sequence of *Streptococcus agalactiae*, a pathogen causing invasive neonatal disease. *Mol. Microbiol* 45 1499–1513. 10.1046/j.1365-2958.2002.03126.x 12354221

[B21] Grissa I. Vergnaud G. Pourcel C. (2007). The CRISPRdb database and tools to display CRISPRs and to generate dictionaries of spacers and repeats. *BMC Bioinformatics* 8:172 10.1186/1471-2105-8-172 PMC1892036 17521438

[B22] Groenen P. M. Bunschoten A. E. van Soolingen D. van Embden J. D. (1993). Nature of DNA polymorphism in the direct repeat cluster of *Mycobacterium tuberculosis*; application for strain differentiation by a novel typing method. *Mol. Microbiol.* 10 1057–1065. 10.1111/j.1365-2958.1993.tb00976.x 7934856

[B23] Haguenoer E. Baty G. Pourcel C. Lartigue M.-F. Domelier A.-S. Rosenau A. (2011). A multi locus variable number of tandem repeat analysis (MLVA) scheme for *Streptococcus agalactiae* genotyping. *BMC Microbiol.* 11:171 10.1186/1471-2180-11-171 PMC3163538 21794143

[B24] Hansen S. M. Uldbjerg N. Kilian M. Sørensen U. B. S. (2004). Dynamics of *Streptococcus agalactiae* colonization in women during and after pregnancy and in their infants. *J. Clin. Microbiol.* 42 83–89. 10.1128/JCM.42.1.83-89.2004 14715736 PMC321715

[B25] Horvath P. Barrangou R. (2010). CRISPR/Cas, the immune system of bacteria and archaea. *Science* 327 167–170. 10.1126/science.1179555 20056882

[B26] Horvath P. Gasiunas G. Siksnys V. Barrangou R. (2013). “Applications of the versatile CRISPR- cas systems,” in *CRISPR-Cas Systems*, eds Barrangou R. van der Oost J. (Berlin: Springer), 267–286. 10.1007/978-3-662-45794-8_11

[B27] Horvath P. Romero D. A. Coûté-Monvoisin A.-C. Richards M. Deveau H. Moineau S. (2008). Diversity, activity, and evolution of CRISPR loci in *Streptococcus thermophilus*. *J. Bacteriol.* 190 1401–1412. 10.1128/JB.01415-07 18065539 PMC2238196

[B28] Imperi M. Pataracchia M. Alfarone G. Baldassarri L. Orefici G. Creti R. (2010). A multiplex PCR assay for the direct identification of the capsular type (Ia to IX) of *Streptococcus agalactiae*. *J. Microbiol. Methods* 80 212–214. 10.1016/j.mimet.2009.11.010 19958797

[B29] Ishino Y. Shinagawa H. Makino K. Amemura M. Nakata A. (1987). Nucleotide sequence of the iap gene, responsible for alkaline phosphatase isozyme conversion in *Escherichia coli*, and identification of the gene product. *J. Bacteriol.* 169 5429–5433. 3316184 10.1128/jb.169.12.5429-5433.1987 PMC213968

[B30] Ivanova N. Daum C. Lang E. Abt B. Kopitz M. Saunders E. (2010). Complete genome sequence of *Haliangium ochraceum* type strain (SMP-2). *Stand. Genomic Sci.* 2 96–106. 10.4056/sigs.69.1277 21304682 PMC3035250

[B31] Jiang F. Doudna J. A. (2015). The structural biology of CRISPR-Cas systems. *Curr. Opin. Struct. Biol.* 30C, 100–111. 10.1016/j.sbi.2015.02.002 25723899 PMC4417044

[B32] Jones N. Bohnsack J. F. Takahashi S. Oliver K. A. Chan M.-S Kunst F. (2003). Multilocus sequence typing system for group B *streptococcus*. *J. Clin. Microbiol.* 41 2530–2536. 10.1128/JCM.41.6.2530-2536.2003 12791877 PMC156480

[B33] Kunin V. Sorek R. Hugenholtz P. (2007). Evolutionary conservation of sequence and secondary structures in CRISPR repeats. *Genome Biol.* 8 R61. 10.1186/gb-2007-8-4-r61 PMC1896005 17442114

[B34] Labrie S. J. Samson J. E. Moineau S. (2010). Bacteriophage resistance mechanisms. *Nat. Rev. Microbiol.* 8 317–327. 10.1038/nrmicro2315 20348932

[B35] Lanotte P. (2012). “Molecular epidemiology of tuberculosis” in *New Frontiers of Molecular Epidemiology of Infectious Diseases*, eds Morand S. Beaudeau F. Cabaret J. (Berlin: Springer Science + Business Media).

[B36] Levin B. R. (2010). Nasty viruses, costly plasmids, population dynamics, and the conditions for establishing and maintaining CRISPR-mediated adaptive immunity in bacteria. *PLoS Genet.* 6:e1001171 10.1371/journal.pgen.1001171 PMC2965746 21060859

[B37] Lopez-Sanchez M.-J. Sauvage E. Da Cunha V. Clermont D. Ratsima Hariniaina E. Gonzalez- Zorn B. (2012). The highly dynamic CRISPR1 system of *Streptococcus agalactiae* controls the diversity of its mobilome. *Mol. Microbiol.* 85 1057–1071. 10.1111/j.1365-2958.2012.08172.x 22834929

[B38] Louwen R. Horst-Kreft D. de Boer A. G. van der Graaf L. de Knegt G. Hamersma M. (2013). A novel link between Campylobacter jejuni bacteriophage defence, virulence and Guillain-Barré syndrome. *Eur. J. Clin. Microbiol. Infect. Dis.* 32 207–226. 10.1007/s10096-012-1733-4 22945471

[B39] Makarova K. S. Haft D. H. Barrangou R. Brouns S. J. J. Charpentier E. Horvath P. (2011). Evolution and classification of the CRISPR- Cas systems. *Nat. Rev. Microbiol.* 9 467–477. 10.1038/nrmicro2577 21552286 PMC3380444

[B40] Makarova K. S. Wolf Y. I. Koonin E. V. (2013). The basic building blocks and evolution of CRISPR-CAS systems. *Biochem. Soc. Trans.* 41 1392–1400. 24256226 10.1042/BST20130038 PMC5898231

[B41] Mayon-White R. T. (1985). The incidence of GBS disease in neonates in different countries. *Antibiot. Chemother.* 35 17–27. 10.1159/000410356 3901893

[B42] Mojica F. J. M. Díez-Villaseñor C. García-Martínez J. Almendros C. (2009). Short motif sequences determine the targets of the prokaryotic CRISPR defence system. *Microbiology* 155 733–740. 10.1099/mic.0.023960-0 19246744

[B43] Mojica F. J. M. Díez-Villaseñor C. García-Martínez J. Soria E. (2005). Intervening sequences of regularly spaced prokaryotic repeats derive from foreign genetic elements. *J. Mol. Evol.* 60 174–182. 10.1007/s00239-004-0046-3 15791728

[B44] Nocard M. Mollereau R. (1887). Sur une mammite contagieuse des vaches laitieres. *Ann. Inst. Pasteur.* 1 109.

[B45] Paez-Espino D. Morovic W. Sun C. L. Thomas B. C. Ueda K. Stahl B. (2013). Strong bias in the bacterial CRISPR elements that confer immunity to phage. *Nat. Commun.* 4 1430 10.1038/ncomms2440 23385575

[B46] Pourcel C. Salvignol G. Vergnaud G. (2005). CRISPR elements in *Yersinia pestis* acquire new repeats by preferential uptake of bacteriophage, DNA and provide additional tools for evolutionary studies. *Microbiology* 151 653–663. 10.1099/mic.0.27437-0 15758212

[B47] Poyart C. Réglier-Poupet H. Tazi A. Billoët A. Dmytruk N. Bidet P. (2008). Invasive group B streptococcal infections in infants, France. *Emerg. Infect. Dis.* 14 1647–1649. 10.3201/eid1410.080185 18826837 PMC2609873

[B48] Poyart C. Tazi A. Réglier-Poupet H. Billoët A. Tavares N. Raymond J. (2007). Multiplex PCR assay for rapid and accurate capsular typing of group B *Streptococci*. *J. Clin. Microbiol.* 45 1985–1998. 10.1128/JCM.00159-07 17376884 PMC1933079

[B49] Price E. P. Smith H. Huygens F. Giffard P. M. (2007). High-resolution DNA melt curve analysis of the clustered, regularly interspaced short-palindromic-repeat locus of *Campylobacter jejuni*. *Appl. Environ. Microbiol.* 73 3431–3436. 10.1128/AEM.02702-06 17400785 PMC1907115

[B50] Salloum M. van der Mee-Marquet N. Domelier A.-S. Arnault L. Quentin R. (2010). Molecular characterization and prophage DNA contents of *Streptococcus agalactiae* strains isolated from adult skin and osteoarticular infections. *J. Clin. Microbiol.* 48 1261–1269. 10.1128/JCM.01820-09 20181908 PMC2849610

[B51] Salloum M. van der Mee-Marquet N. Valentin-Domelier A.-S. Quentin R. (2011). Diversity of prophage DNA regions of *Streptococcus agalactiae* clonal lineages from adults and neonates with invasive infectious disease. *PLoS ONE* 6:e20256 10.1371/journal.pone.0020256 PMC3102099 21633509

[B52] Shariat N. Dudley E. G. (2014). CRISPRs: molecular signatures used for pathogen subtyping. *Appl. Environ. Microbiol.* 80 430–439. 10.1128/AEM.02790-13 24162568 PMC3911090

[B53] Shariat N. Sandt C. H. DiMarzio M. J. Barrangou R. Dudley E. G. (2013). CRISPR-MVLST subtyping of *Salmonella enterica* subsp. enterica serovars Typhimurium and Heidelberg and application in identifying outbreak isolates. *BMC Microbiol.* 13:254 10.1186/1471-2180-13-254 PMC3840669 24219629

[B54] Skoff T. H. Farley M. M. Petit S. Craig A. S. Schaffner W. Gershman K. (2009). Increasing burden of invasive group B streptococcal disease in nonpregnant adults, 1990–2007. *Clin. Infect. Dis.* 49 85–92. 10.1086/599369 19480572

[B55] Stern A. Keren L. Wurtzel O. Amitai G. Sorek R. (2010). Self-targeting by CRISPR: gene regulation or autoimmunity? *Trends Genet.* 26 335–340. 10.1016/j.tig.2010.05.008 20598393 PMC2910793

[B56] Szczepankowska A. (2012). Role of CRISPR/cas system in the development of bacteriophage resistance. *Adv. Virus Res.* 82 289–338. 10.1016/b978-0-12-394621-8.00011-x 22420856

[B57] Tettelin H. Masignani V. Cieslewicz M. J. Donati C. Medini D. Ward N. L. (2005). Genome analysis of multiple pathogenic isolates of *Streptococcus agalactiae*: implications for the microbial “pan-genome.” *Proc. Natl. Acad. Sci. U.S.A.* 102 13950–13955. 10.1073/pnas.0506758102 16172379 PMC1216834

[B58] Touchon M. Charpentier S. Clermont O. Rocha E. P. C. Denamur E. Branger C. (2011). CRISPR distribution within the *Escherichia coli* species is not suggestive of immunity- associated diversifying selection. *J. Bacteriol.* 193 2460–2467. 10.1128/JB.01307-10 21421763 PMC3133152

[B59] van der Mee-Marquet N. Fourny L. Arnault L. Domelier A.-S. Salloum M. Lartigue M.-F. (2008). Molecular characterization of human-colonizing *Streptococcus agalactiae* strains isolated from throat, skin, anal margin, and genital body sites. *J. Clin. Microbiol.* 46 2906–2911. 10.1128/JCM.00421-08 18632904 PMC2546740

[B60] van der Oost J. Westra E. R. Jackson R. N. Wiedenheft B. (2014). Unravelling the structural and mechanistic basis of CRISPR-Cas systems. *Nat. Rev. Microbiol.* 12 479–492. 10.1038/nrmicro3279 PMC4225775 24909109

[B61] Yosef I. Goren M. G. Qimron U. (2012). Proteins and DNA elements essential for the CRISPR adaptation process in *Escherichia coli*. *Nucleic Acids Res.* 40 5569–5576. 10.1093/nar/gks216 22402487 PMC3384332

