# Supplementary material for: Analysis of the type II-A CRISPR-Cas system of Streptococcus agalactiae reveals distinctive features according to genetic lineages
Source: Front Genet. 2015 Jun 15;6:214. doi: 10.3389/fgene.2015.00214 (PMC4466440; doi:10.3389/fgene.2015.00214)
Supplement: Supplementary file 1 [file Table_1.DOCX]

Supplementary Table S1: Primers used for CRISPR1 amplification and sequencing.

| Primer name | Primer sequences | Reference |
| --- | --- | --- |
| *CRISPR1 PCR F* | 5’- GAGACACAGAGCGACACTATC - 3’ | Lopez-Sanchez *et al*., 2012 |
| *CRISPR1 PCR R* | 5’- CATTTCTTTCTCCACTATTATAAC - 3’ | Lopez-Sanchez *et al*., 2012 |
| *CRISPR 1 SEQ F* | 5’- GAAGACTCTATGATTTACCGC - 3’ | Lopez-Sanchez *et al*., 2012 |
| *CRISPR1 SEQ R* | 5’- CAGCAATCACTAAAAGAACCAAC - 3’ | Lopez-Sanchez *et al*., 2012 |
| *Sp4F* | 5’- TGTGTTATCATATCTTCCTTTG - 3’ | This study |
| *Sp39F* | 5’- GCCTTTTCTAACTCTTCAG - 3’ | This study |
| *Sp224F* | 5’- TGCTAAATCATTTATAAATAC - 3’ | This study |
| *Sp246F* | 5’- GATTAGTTTGCGTACTCGCTC - 3’ | This study |
| *Sp275F* | 5’- ACAGACAAAGAAGATGGCAAG - 3’ | This study |
| *Sp298F* | 5’- CTTTTAACCGTCTCTCCGCTTC - 3’ | This study |
| *Sp307F* | 5’- AAGACTTAAAATCGATTAGA - 3’ | This study |
| *Sp340F* | 5’- AACAAGCGCAAAGCTGTC **–** 3**’** | This study |
| *Sp415F* | 5’- AATAATAACGTTGACTTG - 3’ | This study |
| *Sp425R* | 5’- TTTCATCTTCAGTATCA- 3’ | This study |
| *Sp451F* | 5’- GTGATTGTGGGAATACTTGTGG - 3’ | This study |
| *Sp504F* | 5’- TTTATCCCTAGTAGGTT - 3’ | This study |
| *Sp511F* | 5’- GCTTGGGTTTGATAAGGG - 3’ | This study |
| *Sp529F* | 5’- GGTTGGGCTGTTTGTAAG - 3’ | This study |
| *Sp539F* | 5’- TCTAAGTGCTCGACCATC - 3’ | This study |
| *Sp830F* | 5’- TCAAAAGTTTCCACTAATAGCGTTT - 3’ | This study |
| *Sp2027F* | 5’- ACGCTCTAAAATTGGG - 3’ | This study |
| *Sp2122F* | 5’- TCGTAGTATCTGCTATCG - 3’ | This study |
| *Sp177R* | 5’- AGTTACTGTTGAGGGTAGTCC - 3’ | This study |
| *Sp177F* | 5’- AAGGACTACCCTCAACAGTAAC- 3’ | This study |
| *Sp121F* | 5’- ACTAGGAATTGTAGAGATC- 3’ | This study |
| *Sp87R* | 5’- TATGCAAAGCAGTCACCAGC - 3’ | This study |
| *Sp42R* | 5’- TAAGTAACAAGACAGACTTGAA - 3’ | This study |
