# Supplementary material for: Analysis of the type II-A CRISPR-Cas system of Streptococcus agalactiae reveals distinctive features according to genetic lineages
Source: Front Genet. 2015 Jun 15;6:214. doi: 10.3389/fgene.2015.00214 (PMC4466440; doi:10.3389/fgene.2015.00214)
Supplement: Supplementary file 2 [file Table_2.DOCX]

Supplementary Table S2. Inventory and distribution of the nucleotides located at the 3’ end of CRISPR1 leader sequences among *S. agalactiae* clonal complexes or sequence types. Variations (absence, replacement or insertion) as compared to the most frequent leader sequence are indicated in red (absence of a nucleotide is indicated by a red point).

| **Leader sequence** (3’ end) | **Frequency** (%) | **Clonal complex or ST**  (No. of strains) |
| --- | --- | --- |
| …AAAATTTTCTACGAG | 87,3 | CC-1 (25)  CC-10 (12)  CC-17 (30)  CC-19 (13)  CC-23 (26)  ST-4 (1)  ST-388 (2)  ST-24 (1) |
| …AAAATTTTCTACGA. | 10,3 | CC-10 (13) |
| …AAAATTTTCTACGAGGTT | 0,8 | CC-1 (1) |
| …AAAATTTTCTACGAGG | 0,8 | CC- 19 (1) |
| …AAAATTTTCTATGAG | 0,8 | ST-130 (1) |
